# A systematic literature review of time to return to work and narcotic use after lumbar spinal fusion using minimal invasive and open surgery techniques

**DOI:** 10.1186/s12913-017-2398-6

**Published:** 2017-06-27

**Authors:** Xuan Wang, Benny Borgman, Simona Vertuani, Jonas Nilsson

**Affiliations:** 1 Mapi Group, Klarabergsviadukten 90B, SE-111 64 Stockholm, Sweden; 2 Spine & Biologics Medtronic International Trading SARL, Tolochenaz, Switzerland

**Keywords:** Lumbar spinal fusion, Minimal invasive surgery, Open surgery, Transforaminal lumbar interbody fusion, Posterolateral fusion, Posterior lumbar interbody fusion, Anterior lumbar interbody fusion, Systematic literature review, Return to work, Narcotic use

## Abstract

**Background:**

Chronic low back pain is a common health problem for adult workers and causes an enormous economic burden. With the improvement of minimally invasive surgical techniques (MIS) in spinal fusion and the development of fusion devices, more lumbar operations are today being performed through a less invasive technique. When compared with open surgeries (OS), MIS has demonstrated better clinical outcomes including operation time, blood loss, complication rates and length of hospital stay. The aim of this review was to identify and summarize evidence on the time to return to work and the duration of post-operation narcotic use for patients who had lumbar spinal fusion operations using MIS and OS techniques.

**Methods:**

A systematic literature review was performed including studies identified from PubMed, EMBASE, the Cochrane Collaboration, and the Centre for Review and Dissemination (CRD) (January 2004–April 2014) for publications reporting on time to return to work and post-operation narcotic use after MIS or OS lumbar spinal fusion surgeries.

**Results:**

Out of a total of 36 included studies, 28 reported on the time to return to work and 17 on the narcotic use after MIS or OS. Four studies described the time to return to work directly comparing MIS and OS. Three studies, from the US, directly compared the duration of narcotic use between MIS- transforaminal lumbar interbody fusion (TLIF) and OS-TLIF. In addition to the time to return to work, 23 studies reported on the rate of return to work and the employment rate before and after surgery, and two Swedish studies presented sick leave data.

**Conclusions:**

There is a gap of good quality data describing the time to return to work and narcotic use after lumbar spinal fusion operations using MIS or OS techniques. However, the current systematic literature review indicates that patients who have lumbar spinal fusion operations, with the MIS procedure, generally return to work after surgery more quickly and require less post-operation narcotics for pain control compared to patients who have OS.

**Electronic supplementary material:**

The online version of this article (doi:10.1186/s12913-017-2398-6) contains supplementary material, which is available to authorized users.

## Background

Chronic low back pain is a leading common health problem for adult workers worldwide [1, 2]; it is the leading cause of activity limitation, job-related disability and absence from work, and it causes an enormous economic burden [3].

The Global Burden of Disease study [3] estimated that low back pain causes more global disability than any other condition. According to its estimates in 2010, the total costs of the condition in the United States exceed $100 billion annually, with two-thirds of these costs come from lost wages and decreased work productivity [4, 5].

Surgical treatment of the lumbar spine has been shown to be effective in reducing patient’s pain and improving function and disability relative to non-surgical treatment [6]. In addition, surgical treatment has been proven to be cost-effective over a 4-year period compared with none-surgical care [7].

A recent study examined the effects of lumbar spinal surgery on work productivity with regard to earnings and absence from work and concluded that reduced productivity losses, after disc herniation surgery, may offset the increased direct medical costs associated with surgery [8]. Lumbar spinal fusion surgery is a viable treatment option for reducing pain and improving function in patients with chronic pain refractory to non-surgical care [9].

Minimally invasive surgery (MIS) techniques for lumbar spinal fusion are equivalent to traditional open surgery (OS) procedures in terms of post-operation fusion rates [10], while MIS has the advantage of reducing tissue damage to the spinal muscles compared to OS [11].

The use of MIS techniques in lumbar spinal surgery has increased as improved patient outcomes and lower hospital costs have been recognized [12–14].

Several studies have demonstrated short term benefits of MIS such as rapid mobilization, shorter length of hospital stay, reduced blood loss, less post-operation pain, reduced risk of infection, and reduced need for post-operation analgesics [15]. Better clinical outcomes compared to OS have been described for a number of different incision approaches such as transforaminal lumbar interbody fusion (TLIF), posterior lumbar interbody fusion (PLIF) and anterior lumbar interbody fusion (ALIF) [16, 17].

Parker et al. showed that MIS-TLIF was associated with reduced costs over 2 years with similar health utilities as OS-TLIF [18] and, MIS-TLIF lumbar spinal fusion resulted in a statistically significant reduction in total hospital costs [14]. However, the economic evaluations of MIS and OS paid little attention to the societal perspective related to initial return to work and productivity after MIS surgery. In addition, earlier narcotic independence following lumbar spinal fusion is another factor that may influence the ability to return to normal work activities.

This systematic literature review (SLR) focused on identifying evidence from published literature on time to return to work and post-operation narcotic use after lumbar spinal fusion operations using MIS or OS techniques. The main objective with the study was to determine whether there is evidence supporting the hypothesis that early post-operation benefits of MIS, compared to OS, have an effect on work productivity.

## Methods

A literature review protocol was developed to design the systematic review detailed search strategies, criteria for study selection, and outcomes to be reported. The PRISMA 2009 checklist (Preferred Items for Systematic Reviews and Meta-Analyses) was used for reporting this review.

### Data sources

The following electronic databases were searched from January 1, 2004 to April 22, 2014: National Library of Medicine’s online PubMed, EMBASE, the Cochrane Collaboration, and the Centre for Review and Dissemination (CRD). The search strategy for PubMed is available as Supplementary material (Additional file 1: Sample search strategy). In addition, evidence was obtained online from the following HTA agencies:

- National Institute for Health and Clinical Excellence (NICE)
- Pharmaceutical Benefits Advisory Committee (PBAC)
- Canadian Agency for Drugs and Technologies in Health (CADTH)

Conference proceedings from meetings organized by the following associations were also searched:

- International Society for Pharmacoeconomics Outcomes Research (ISPOR)
- European Federation of National Associations of Orthopaedics and Traumatology (EFORT)
- American Academy of Orthopaedic Surgeons (AAOS)
- Orthopaedic Trauma Association (OTA)
- North American Spine Society (NASS)
- International Society for the Advancement of Spine Surgery (ISASS)
- Society for Minimally Invasive Spine Surgery (SMISS)

A manual review of the bibliographies of identified key publications was also conducted in order to identify any relevant publications that were not identified through the electronic database searches.

### Study eligibility

Only studies that fulfilled the inclusion criteria, guided by the PICOS approach [19], were included in the study:

- Published articles, posters, abstracts, reports and conference proceedings
- Studies focused on diseases that need to be treated with lumbar spinal fusion surgery using OS and MIS procedures
- All types of MIS and OS techniques were considered
- Studies of time to return to work, studies on adult population who were not retired and suffering from low back pain and had undergone spinal fusion
- Studies on post-operation narcotic use with no restriction of patients’ age

Studies comparing the time to return to work following lumbar spinal fusion with MIS or OS were of primary interest. The following exclusion criteria were used for the retrieved studies:

- Time to return to work studies including patients of age < 18 or >65 years
- Languages other than English, German, and Swedish
- Surgical technique (i.e., MIS or OS) not specified
- Study objective of narcotic use focused on peri-operation rather than post-operation narcotic usage
- Comparisons of different routes of narcotics administration
- Articles published prior to 2004

### Study selection

Studies identified through the searches in PubMed, EMBASE, Cochrane, and CRD were electronically stored in a Reference Manager database (version 12) and entered into an Excel file which facilitated conduction and tracking of selection process. The study selection was completed through two levels of review by two independent reviewers: the Level I review involved scanning of titles and abstracts and the Level II review involved scanning the full text of articles which had not been excluded in the Level I. Any discrepancies between the two reviewers were resolved by a third reviewer.

### Data extraction

Data were extracted on study characteristics, patient information, surgery information, return to work outcomes, narcotic usage, and economic outcomes by a single reviewer. To ensure the validity of the extracted data, studies were randomly quality checked by a second reviewer who did not perform the initial extraction. Any disagreements were resolved through discussion, or by a third party, until consensus was reached.

### Quality assessment

To account for any bias and uncertainty within the publications, included studies were assessed for methodological rigor and quality using the NICE methodology checklist, as set out in the NICE technical manual “Methods for the development of NICE public health guidance (third edition)” [20]. 

## Results

**Fig. 1:**
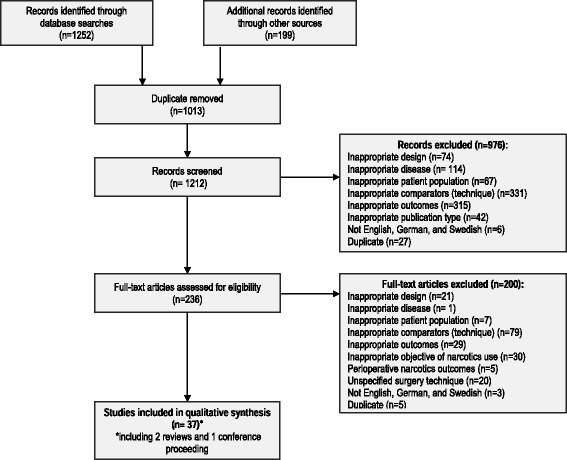
Flowchart detailing the review process

A total of 1212 unique records were obtained from all the searches; of which, 199 were retrieved from HTA agencies and conference proceedings. Out of these, 236 were selected for full text evaluation and, eventually, 36 studies were included for full text review and data-extraction. The selection process and the number of articles included at each consecutive step of the SLR are described in Fig. 1. The complete list of the included articles is available as supplementary material (Additional file 2: List of included studies).

### Characteristics of included studies

Studies were undertaken in a range of 13 countries, where the majority was found from the US (*n* = 16) followed by South Korea (*n* = 5) and Sweden (*n* = 3). Among the 36 included studies, 28 reported outcomes on the time to return to work and 17 on the use of narcotics after MIS or OS. Out of the 28 studies reporting time to return to work, more than half (*n* = 18) were observational studies, four were randomized controlled trials (RCTs), and five were economic evaluation studies. Only five studies directly compared the time to return to work between MIS and OS and none of them were RCTs. A majority of the studies reporting on post-operation narcotic use were observational studies (*n* = 13) and ten studies directly compared MIS and OS.

**Fig. 2:**
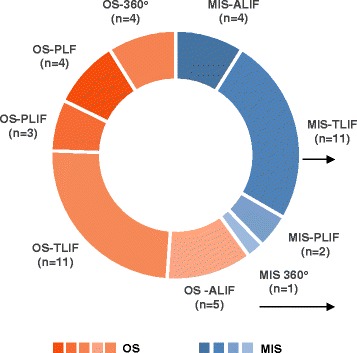
Surgical procedures performed in the studies included in the review. “360 procedure”: Posterolateral fusion plus internal fixation with the variable screw placement device plus interbody fusion; ALIF: Anterior Lumbar Interbody Fusion; MIS: Minimal Invasive Surgery; OS: Open Surgery; PLF: Posterolateral Fusion; PLIF: Posterior Lumbar Interbody Fusion; TLIF: Transforaminal Lumbar Interbody Fusion

Out of the 21 studies reporting on MIS, TLIF was used in 11 studies, followed by ALIF (*n* = 4) and PLIF (*n* = 2). In the studies focusing on OS, TLIF was also found to be the most frequently studied technique (*n* = 11), followed by ALIF (*n* = 5), “360° procedure” (i.e., posterolateral fusion plus internal fixation with the variable screw placement device plus interbody fusion) (*n* = 4) and PLIF (*n* = 4). The different surgical procedures performed in the studies included in the review are described in Fig. 2.

**Fig. 3:**
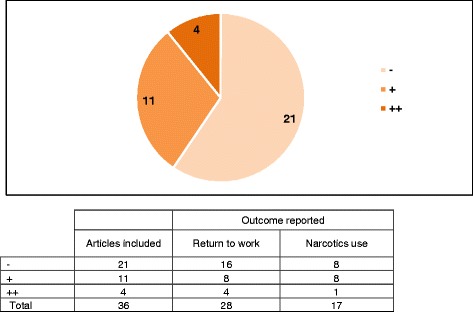
Quality assessment of the included studies. ++ All or most of the criteria have been fulfilled. Where they have not been fulfilled the conclusions of the study or review are thought very unlikely to alter. + Some of the criteria have been fulfilled. Those criteria that have not been fulfilled or not adequately described are thought unlikely to alter the conclusions. - Few or no criteria fulfilled. The conclusions of the study are thought likely or very likely to alter

Only four studies fulfilled most criteria of the NICE quality assessment checklist (++) and more than half (*n* = 21) only fulfilled a few criteria (Fig. 3). The reason for this is that most observational studies had low representativeness (e.g., a small sample size, single centre study, and only one or two surgeons performed the surgeries). In addition, in some cases the study methods and results were not sufficiently described and/or the study questions were not clearly addressed. A table of the assessment results per included article is available as supplementary material (Additional file 3: Quality assessment results using the NICE methodology checklist).

### Return to work after lumbar spinal fusion surgery

The 28 studies including data on return to work reported results differently. Several studies estimated the actual time (i.e., the number of weeks or months) to return to work after surgery, while most studies reported the proportion of patients returning to work at different follow-up intervals after surgery. The relevant studies have been divided into three groups: i) studies that directly compared MIS and OS; ii) studies that used MIS; or iii) OS techniques (Table 1).

**Table 1:** Results on time to return to work and rate of return to work

Study	Country	Number of patients (Technique)	Fusion levels	Follow up	Time to return to work Mean (weeks)		Return to work Rate (%)	
					MIS	OPEN	MIS	OPEN
*MIS* vs. *OS studies*								
Adogwa et al. (2011) [21]	US	15 (MIS-TLIF)15 (OS-TLIF)	1-level	2 years	Median: 8.5 [IQR: 4.4–21.4]	Median: 17.1 [IQR: 1.7–35.9]	At 2 years: ~90%	At 2 years: ~80%
Kim et al. (2010a) [23]	South Korea	43 (MIS-ALIF) 32 (OS-ICF)	NR	41.1 months (MIS) 32.9 months (OS)	~14.8 (fulltime work)	~14.4 (fulltime work)	-	-
Parker et al. (2012) [18]	US	15 (MIS-TLIF)15 (OS-TLIF)	1-level	2 years	8.3	16.3	**-**	**-**
Parker et al. (2013) [22]	US	50 (MIS-TLIF) 50 (OS-TLIF)	1-level	2 years	Median: 7 [4–11]	Median: 11 [9–16]	At 6 months: ~95%	At 9 months: ~80%
*MIS studies*								
Eckman et al. (2012)^a^ [32]	NR	606 (MIS-TLIF)	1	NR	-	-	96%	-
Eckman et al. (2014) [31]	US	1005 (MIS-TLIF)	1- or 2-level	3 months	-	-	96% of procedures (same-day discharge group)93% of procedures (hospital stay group)	-
Kim et al. (2009) [24]	South Korea	48 (MIS-ALIF) 46 (MIS-TLIF)	NR	32.6 months (ALIF) 29.7 months (TLIF)	6.1 months (ALIF)10.9 months (TLIF)	-	-	-
Rouben et al. (2011) [25]	US	169 (MIS-TLIF)	1-level (124)2-level (45)	49 months	11 weeks (for 97% RTW right after surgery)17 weeks (for 57% compensation workers)	-	97% RTW right after surgery 57% compensation workers RTW	-
Kim et al. (2010b) [35]	South Korea	63 (MIS-ALIF)	NR	6 years	-	-	83%	-
Kim et al. (2012) [34]	South Korea	44 (MIS-ALIF)	1-level	71.8 months (DS) 66.4 months (IS)	-	-	91%	-
Zeilstra et al. (2013) [33]	US	131 (MIS-AxiaLIF)	1-level	21.8 months	-	-	64%44% (for fulltime workers)	-
*OS studies*								
Berg et al. (2009) [36]	Sweden	72 (OS-PLF/PLIF)	1-level (33)	2 years	-	-	-	72.0%
Corenman et al. (2013) [37]	NR	36 (OS-TLIF)	1-level (24)2-level (12)	41.9 months	-	-	-	84.4%
Fritzell et al. (2004) [29]	Sweden	72 (OS-PLF) 71 (OS-VSP)74 (OS-"360”)	NR	2 years	-	-	-	33%
Gornet et al. (2011) [28]	US	172 (OS-ALIF)	1-level	2 years	-	96 days	-	-
Robertson et al. (2004) [26]	New Zealand	35 (OS-PLF)	1-level (12)2-level (16)	31 months	-	3–50 months	-	100%
Takahashi et al. (2011) [27]	Japan	21 (OS-TLIF)	1-level (18)2-level (3)	26.1 months	-	-	-	90% after 3.9 months

“360 procedure”: Posterolateral fusion plus internalfixation with the variable screw placement device plus interbody fusion; ALIF: Anterior Lumbar Interbody Fusion; AxiaLIF: Axial Lumbar Interbody Fusion; DS: Degenerative Spondylolisthesis; ICF: Instrumented Circumferential Fusion; IQR: InterQuartile Range; MIS: Minimal Invasive Surgery; NR: Not Reported; OS: Open Surgery; PLF: PosteroLateral Fusion; PLIF: Posterior Lumbar Interbody Fusion; PSG: Prior Surgery Group; RTW: Return To Work; TLIF: Transforaminal Lumbar Interbody Fusion; VSP: Posterolateral fusion plus internal fixation with the variable screw placement device

^a^Published as abstract

### Time to return to work

#### MIS vs. OS studies

Four studies [18, 21–23] were identified that directly compared the time to return to work after lumbar spinal fusion with the MIS and OS procedures. Out of these, three [18, 21, 22] have been conducted in the US by the same research group. Adogwa et al. [21] and Parker et al. [18] reported the time to return to work after of MIS-TLIF or OS-TLIF in a pilot study including 15 patients per group, while Parker et al. [22] studied a larger cohort of 100 patients (50 patients per MIS-TLIF or OS-TLIF group) with a follow-up period of 2 years. This study confirmed the trend from the earlier study [18] where it was observed that patients who had MIS-TLIF returned to work after surgery, significantly earlier compared to patients who had OS-TLIF (7 vs. 11 weeks, *p* = 0.02).

Kim et al. [23] examined two cohorts of patients who underwent MIS-ALIF or OS-circumferential fusion at two different hospitals. The mean time to return to work after surgery was 3.7 months for patients in the MIS-ALIF group and 3.6 months in the OS-instrumented circumferential fusion group. The difference was not statistically significant.

#### MIS studies

Five studies [24–28] reported results on the time to return to work for only MIS or only OS procedures used, thus not comparing the two techniques side by side. Kim et al. [24] retrospectively analysed clinical data from 48 patients who had instrumented MIS-ALIF and 46 patients who had instrumented MIS-TLIF. The mean follow-up period was more than 2 years for both groups and the mean time to return to work was found to be 6.1 months in the ALIF group and 10.9 months in the TLIF group (*p* = 0.0188).

Rouben et al. [25] retrospectively studied 169 patients who had MIS-TLIF, in which 45 patients had two-level spinal fusion. Compared to the results from Kim et al. [24], Rouben et al. [25] found a much shorter time to return to work after surgery; the mean time for the patients who were working immediately before surgery was 11 weeks (median: 8 weeks), while a slightly longer time to return to work (mean: 17 weeks) was observed for patients with work compensation. In addition, about 91% of all patients were discharged from hospital within 24 h after surgery and the longest hospital stay was 3 days. Note that this study was carried out in a single centre in the US where MIS surgery was their primary standard of practice and OS was not often carried out. Kim et al. [24] concluded that, the significant difference in the time to return to work between MIS-ALIF and MIS-TLIF may be due to the difference between the surgical techniques and the mean time to return to work (here it refers to the time to a return to full and unrestricted activity rather than the initial return to work after surgery).

#### OS studies

Three studies [26–28] focused on only OS. A randomized controlled investigational device exemption trial conducted by Gornet et al. [28] compared 405 patients treated with lumbar disc arthroplasty with a control group of 172 patients who received OS-ALIF. The median time to return to work was 96 days after OS-ALIF surgery.

A small cohort study by Takahashi et al. [27] reported that 18 of 20 (90%) patients who had worked before OS-TLIF surgery returned to work after an average of 3.9 months. The time to return to work for patients in the heavy labour group was longer than patients in the light labour group (5.0 months vs. 3.2 months).

In another study, Robertson et al. [26] investigated 35 patients who had one- or two-level posterolateral fusion (PLF) or PLIF over a 5-year period. Among the 28 patients who were available for follow-up at review, 23 patients were covered by New Zealand’s Accident Compensation System and five were not. All five patients with no compensation showed much shorter time to return to work after surgery (average of 3 months, SD not reported) while patients with full compensation had the longest time to return to work (average of 50 months, SD not reported).

### Rate of return to work

A number of studies did not indicate at what time point patients returned to work following surgery; instead, estimates of the rate of return to work were reported. In addition, among the studies only focused on OS, seven compared the employment rate before and after surgery and two studies [29, 30] described the number of sick leave days after surgery.

#### MIS vs. OS studies

In terms of the rate of return to work, Adogwa et al. [21] and Parker et al. [22] also provided direct comparison between OS-TLIF and MIS-TLIF during 2 years follow-up. Both studies observed that more patients who were treated with MIS-TLIF returned to work, at each follow-up time, compared to OS-TLIF patients. Furthermore, more than 90% of the patients who received MIS returned to work within 12 months and over 80% of the patients who had OS returned to work within 12 months after surgery.

#### MIS studies

Six studies [25, 31–35] reported the rate of return to work for only MIS. In Eckman et al. [31], the same-day discharge cohort consisted of a total of 728 patients who had 808 MIS-TLIF procedures and the hospital stay cohort included 277 patients who had 306 MIS-TLIF procedures. The authors reported that, after 3 months, the rate of return to work were 96% of procedures in the same-day discharge group and 93% of procedures in the hospital stay group. In an abstract retrieved from Eckman et al. [32], a total of 394 patients who had MIS-TLIF were working within 30 days prior to surgery and 96% of patients (367 out of 384 patients with data available) returned to work after surgery, but the follow-up period was not provided.

In Rouben et al. [25], 97% of patients who worked immediately before surgery and 57% of patients who had work compensation had returned to work during the 4 years after surgery. Zeilstra et al. [33] retrospectively studied a total of 131 patients who were treated with one-level MIS-axial lumbar interbody fusion (AxiaLIF) with at least 1 year follow-up (mean: 21 ± 8 months). The rate of patients who returned to work was not reported. Instead, the authors observed that the employment rate increased from 47% before surgery to 64% at final follow-up (pre-operation vs. post-operation: *p* < 0.001), and the employment rate of full-time work increased from 24% to 44% after surgery (*p*-value not reported). Kim et al. [35] found that 83% of patients who had MIS-ALIF returned to work within 72 months mean follow-up. Not all patients were able to return to same work or job/level of employment, and about half of them (47.1%) had to change work or level of job (37.7%) or even part-time work (9.4%). A total of 44 patients who had MIS-TLIF were studied by Kim et al. in 2012 [34] and over 90% of patients who were working before the surgery returned to work within 70 months mean follow-up, and only one patient received work compensation.

Overall, about 83%–97% of patients who were working before surgery were able to return to work within 6 years [25, 31–35]. Although the follow-up intervals varied across these studies, a high rate (93%–96% of procedures) of patients who returned to work after MIS was observed after the first 3 months [31, 32] and it was not always clear what kind of jobs the patients had. Only Kim et al. [35] reported that a substantial amount of patents had to change work after surgery (47.1%).

#### OS studies

Five studies [26, 27, 29, 36, 37] included data on the rate of return to work after OS surgery. Berg et al. [36] reported on a clinical RCT comparing total disc replacement (TDR) and instrumented lumbar spinal fusion (i.e., PLF or PLIF) in Sweden. The clinical outcomes of both groups (TDR: *n* = 80 vs. fusion: *n* = 72) were improved after the operation. After 2 years, 76% of patients in the fusion group were back to part-time or full-time work.

In another Swedish study, conducted by Fritzell et al. [29], a total of 284 patients were studied who were randomized to either lumbar spinal fusion (*n* = 217) or a non-surgical control group (*n* = 67). Patients who were receiving surgeries were divided into three groups. The authors found that after 2 years: 35% of the patients who had PLF, 34% of the patients who had PLF plus internal fixation with the variable screw placement device (VSP) and 39% of the patients who had PLF plus VSP and interbody fusion (“360 procedure”) had returned to work. The difference between the groups may partly be explained by the surgical fusion techniques used.

Corenman et al. [37] presented a small, 2 year retrospective observational study, where data were collected from a return to work questionnaire. The study found that 84.4% (27/32) of the cohort were able to return to the same job as before the surgery. In another small retrospective study with a 1-year minimum follow-up period, Takahashi et al. [27] reported that 90% of the patients (18 of 20) returned to work and 59% (10 of 17) had to reduce working hours or change work duties. Robertson et al. [26] reported that the number of patients who were on full compensation (i.e., not working) was reduced by 75% after surgery (pre-operation: *n* = 16 vs. post-operation: *n* = 4).

Overall, the studies focused on OS [26, 27, 29, 36, 37] including PLF, PLIF, and TLIF, as well as circumferential fusion techniques reported a greater variation of rate of return to work (range from 18% to 100%) within 3 months to 4 years compared to MIS studies.

Eight of the studies that focused on OS only described the employment rate both pre-operation and post-operation. Overall, a clear trend was observed that the employment rate was increased at 6 months after surgery. Results are summarized in the Supplementary material (Additional file 4: Results of pre-operation vs. post-operation employment rate). In three studies [28, 38, 39] including patients who had ALIF fusion, fairly consistent findings were reported; the employment rate pre-operation ranged from 56% to 58%, while the employment rate 6 months post-operation ranged from 63% to 73%. In Fritzell et al. [30] and Guyer et al. [40] including patients who received PLF or PLIF lumbar spinal fusion operations, the employment rates were 30% and 56% before surgery, and after surgery the employment rates were 72% and 47%, respectively. It should be noted that the employment rate for patients working part-time was not reported in Guyer et al. [40], while 47% of the patients worked full-time.

Two Swedish cost-effectiveness studies [29, 30] reported on sick leave for patients who had OS. The number of days on sick leave during a 2-year period after surgery was collected from the Swedish Social Insurance Board. In the first study, Fritzell et al. [29] reported a total of 521 sick leave days per patient who had OS and, in the second study, Fritzell et al. [30] reported a total of 252 sick leave days per patient who had PLF or PLIF during the first 2 years after the operation. Although a relatively high employment rate (i.e., 72% working full- or part-time) was observed after surgery in Fritzell et al. [30], patients who returned to work after surgery had substantial recurrent sick leave or rehabilitation episodes. The number of levels fused was not reported in either of the studies.

### Return to daily activities or full function

**Fig. 4:**
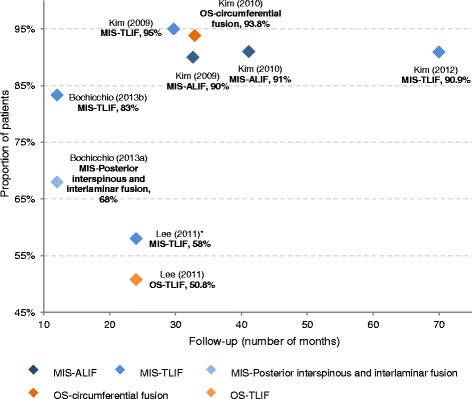
Proportion of patients return to normal activities or full function. *Results of proportion of patients return to full function in Lee et al. (2011) [43]. ALIF: Anterior Lumbar Interbody Fusion; MIS: Minimally Invasive Surgery; OS: Open Surgery; TLIF: Transforaminal Lumbar Interbody Fusion

In addition to time and rate of return to work, six studies [23, 24, 34, 41–43] reported the number of patients who returned to full function or normal daily activities after lumbar spinal fusion using MIS or OS during follow-up. Overall, 68%–95% of patients who had MIS returned to daily activities within the mean follow-up of 12 to 70 months after surgery [24, 34, 41, 42] and 58% of patients return to full function at 2 years in Lee et al. [43], as illustrated in Fig. 4. Higher rates were usually observed at longer follow-up periods. In addition, Kim et al. [23] reported higher rates in return to daily activities for patients who had OS-instrumented circumferential fusion (93.8%) versus MIS-ALIF (91%) after about 3 years post-operation, but the difference was not significant.

### Post-operation narcotic use after spinal fusion surgery

Out of 17 studies, 11 directly compared MIS and OS in terms of post-operation narcotic use; however, only three studies [18, 21, 22] reported on time to narcotic independence and the remaining studies [18, 21, 22, 25, 26, 28, 33, 38, 43–52] presented various results.

### Duration of post-operation narcotic use

**Fig. 5:**
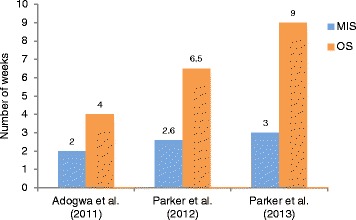
Duration of post-operation narcotic use (MIS vs. OS)

Three studies directly compared the time to narcotic independence between MIS and OS (Fig. 5). Adogwa et al. [21] and Parker et al. [18] reported that the length of narcotic use after surgery was, significantly, shorter for patients who underwent MIS-TLIF (median: 2 weeks [21]; mean: 2.6 weeks [18]) compared with OS-TLIF (median: 4 weeks [21]; mean: 6.5 weeks [18]; MIS vs. OS: *p* = 0.008 [18, 21]). In Parker et al. [22], patients in the MIS-TLIF group had threefold shorter time on narcotic use, post-operation, than patients in the OS-TLIF group (median: 3 weeks vs. 9 weeks); however, this substantial difference did not reach statistical significance (*p* = 0.14) due to great variability in the post-operation narcotic use for both cohorts.

### Other results of post-operation narcotics

In addition to the duration of post-operation narcotic use, a number of studies reported results reflecting other post-operation narcotic uses (Additional file 5: Other results of post-operation narcotic usage). A total of ten studies [18, 21, 22, 43, 44, 46, 48–51] directly compared the MIS and OS procedures, three studies [25, 33, 52] only focused on MIS and five studies only used OS [26, 28, 38, 45, 47]. The proportion of patients who used narcotics for pain control, at different follow-up intervals were frequently described, but the definition of narcotics was not clearly described. Although various approaches were used to present the outcomes of post-operation narcotic use, the results were favourable for the MIS technique compared to the OS across all these studies.

## Discussion

With the improvement of MIS surgical techniques in spinal fusion and the development of fusion devices, more operations are today being performed using MIS techniques. A number of previous studies [16, 17] have demonstrated that MIS techniques for lumbar spinal fusion have improved the clinical outcomes including operation time, blood loss, complication rates and length of hospital stay, resulting in less hospital resource utilization; however the benefits of MIS techniques such as faster return to work and productivity which means reducing indirect costs to patients and society are less explored. This SLR was conducted to identify and summarize evidence on the time to return to work and the duration of post-operation narcotic use for patients who had lumbar spinal fusion operations using the MIS and OS techniques. Compared with a previous literature review, published by Parker et al. in 2012 [53], this SLR has a broader scope of time to return to work (e.g., time, rate, employment status, and sick leave) and less restrictions on the type of surgical technique that were used (e.g., TLIF or PLIF).

Out of a total of 36 included studies (including five abstracts [32, 41–43, 46]), two thirds of the studies (*n* = 25) were observational studies and only five studies [28, 36, 38, 40, 48] were RCTs. This might to some extent reflect the actual situation in surgical research, i.e., retrospective case series are more commonly used, usually with small cohorts, while RCTs are more widely used for pharmacological therapies [54, 55]. TLIF was found to be the most frequently studied technique for lumbar spinal fusion operations regardless if MIS or OS was used. Twenty-seven studies described the position of the fusion or the number of levels that were fused for the study patients; out of which, nine studies [25–27, 31, 32, 37, 47, 56, 57] included patients who had two or more levels fused (range from 14% - 57% of total studied patients), but the results were not presented separately by fusion levels. Therefore, the current SLR mainly provides evidence of the time to return to work and the post-operation narcotic use for patients after a single-level lumbar spinal fusion.

The current SLR identified four studies [18, 21–23] that directly compared the time to return to work for patients who had lumbar spinal fusion with the MIS and OS procedures. The three US studies [18, 21, 22] showed almost half the time to return to work for patients who had MIS-TLIF (range of absolute mean: 7.0 to 8.5 weeks) compared to patients who had OS-TLIF (11.0 to 17.1 weeks). Additionally, more than 80% of the patients who received MIS-TLIF or OS-TLIF returned to work within 12 months after surgery [21, 22]. When patients who had MIS-ALIF were compared with OS-instrumented circumferential fusion, Kim et al. [23] reported similar times to return to work for both procedures (3.7 vs. 3.6 months), but no statistically significant difference. It should be noted that two different surgical fusion techniques have been performed at separate hospitals which may explain the lack of difference. Furthermore, the mean follow-up period was 8 months shorter for patients in the OS group compared to the MIS group (32.9 vs. 41.1 months). In this Korean study [23] the surgical techniques were different from the three US studies [18, 21, 22] and a stringent definition of return to work was applied: “return to full and unrestricted activity”, as a result, the time to return to work for both the MIS and the OS groups were different from the results presented in the US studies [18, 21, 22], potentially reflecting cultural practices in Korea. In Kim et al. [24], the time to return to full and unrestricted activity were also used and, therefore, longer times to return to work have been presented compared to the US studies for the MIS groups [18, 21, 22]. Another US study, conducted by Rouben et al. [25], reported a similar time to return to work (11 weeks) as the US studies [18, 21, 22] for patients who had MIS-TLIF. An US study by Gornet et al. [28] focused on only OS-ALIF reported time to return to work with 96 days (13.7 weeks). Based on the findings from these US studies [18, 21, 22, 28], it can be concluded that MIS patients return to work faster compared to OS patients.

The NICE checklist was followed for quality assessment in this review; in general the quality of the included studies was poor. Only four studies [28, 29, 38, 40] fulfilled most criteria and many of the included studies (*n* = 21) only fulfilled few or very few criteria, e.g., the patient samples were small ranging from eight to 72 patients per treatment arm, and were often from a single institution treated by only one or two surgeons. Clearly, there is a gap of evidence around how the time to return to work after lumbar spinal fusion operations differs between the MIS and OS techniques. The interpretation of the current evidence suggests that the time to work after MIS is shorter than after OS operations.

In addition to the time to return to work, several studies were found that reported on the rate of return to work and the employment rate before and after surgery. For patient who had MIS, 83%–97% return to work after surgery within 3 months to 6 years. For patients who had OS a greater variation in the share of patient who returned to work was observed ranging from 18% to 100% within 3 months to 4 years depending on the techniques being used, i.e., PLF, PLIF, TLIF and circumferential fusion. Information regarding the type of jobs the patients was working with before and after the surgery was limited. Kim et al. [35] and Takahashi et al. [27] reported that a substantial amount of patients had to change work or reduce working hours after surgery (47.1 and 59%, respectively).

With regard to the employment rate pre- and post-operation, the combined employment rate of full-time and part-time work was frequently reported. Studies focused on OS only in general showed a clear trend of increased employment rate after surgery. Three studies [28, 38, 39] that focused on OS-ALIF reported fairly consistent time to return to work of 6 months. The employment rate pre-operation ranged from 56% to 58%, while the employment rate 6 months post-operation ranged from 63% to 73%. In addition, only two Swedish cost-effectiveness studies [29, 30] were found that reported on sick-leave for patients who had lumbar spinal fusion with OS; no information on sick-leave was found for MIS in this SLR.

Six studies [23, 24, 34, 41–43] were found describing the return to normal daily activities or full function post-operation. However, the definition of return to daily activities and full function was, generally, not clearly described.

Various factors may affect the return to work, which have not been widely and consistently assessed across the identified studies. Several studies reported the work status (e.g., full-time or part-time), type of work (e.g., heavy or light labour work) and workers’ compensation status prior to surgery led to different return rate or time duration after MIS or OS [26, 27, 29, 37, 38]. In addition, most studies reported patients’ clinical characteristics (e.g., blood loss, surgery time, interbody fusion technique and number of fusion levels), which may affect the return to work. Furthermore, surgery-related and neurological complications may be associated with the return to work. However, due to the small sample size and few observed complications, these factors have not been sufficiently studied [22, 43, 49, 51]. Recent reviews, in other conditions [58, 59], have reported that age, sex, education and other social-economic factors are important factors affecting the return to work, which could be investigated in future studies on MIS or OS in spinal infusion.

Altogether the results of time to and rate of return to work, and comparisons of pre- and post-operative employment rates show benefit for MIS compared to OS. A review of cost of illness studies on chronic low back pain [60] has showed that the direct cost associated with low back pain accounted for only 22% of the total costs, which indicated that the indirect cost caused by the loss of productivity contributed with a much larger share of the overall cost for chronic low back pain. The possibility to return to work faster after surgery and be productive may be one of the largest societal advantages of MIS compared to OS. Reduced absenteeism from work is not only important for the societal costs but also for patients’ quality of life; patients who are able to faster return to normal daily activities are more likely to recover faster due to effective relief of symptoms and disability [61, 62].

Post-operation narcotic use was at focus in 17 studies; out of which 11 studies directly compared MIS versus OS, but again only the three US studies [18, 21, 22] reported the duration of post-operation narcotic use. Similar results as for the time to return to work, MIS-TLIF was associated with half the period of narcotic use after surgery (range of absolute mean time of post-narcotic use: 2–3 weeks) compared to OS-TLIF (mean range: 3–9 weeks). The difference was statistically significant in Adogwa et al. [21] and Parker et al. [18] but not in Parker et al. [22] where patients in the OS-TLIF group had threefold longer narcotic use post-operation than MIS-TLIF (median: 3 vs. 9 weeks). It is likely that the advantages of the MIS procedure (e.g., less muscle damage) contributed to the shorter time of narcotic use during the recovery period after surgery. Duration of narcotic use after surgery may be affected by pre-operation narcotic uses, because patients who used narcotics pre-operation are more likely to continue their pain medications after surgery or require time to quit the narcotics due to, for example, rebound effect; however, the duration of pre-operation narcotic use was not available in these studies. No significant difference in the use of narcotics post-operation between MIS and OS was found in most studies that directly compared MIS to OS, but a majority of studies indicated less use of narcotics for patients after MIS.

Chronic back pain in the lumber region is one of leading cause of disability and there is a high incidence of psychiatric comorbidities (e.g., depression) [63] and substance abuse (narcotics or other drugs) [64] among patients with degenerative disc disease. None of the studies discussed the psychiatric situation of the patient and the abuse of narcotics among patients with disc degenerative disorders. Furthermore, most studies were observational studies and the decision to carry out OS or MIS spinal fusion operations often depended on surgeon’s proficiency and preference.

The post-operation narcotic use was not clearly, and consistently, defined across studies which limit the possibility to compare the results. Just as the time to return work, the narcotic use following spinal surgery operation is a likely differentiator between the MIS and OS procedures. Therefore, future clinical trials would benefit from more focus on the narcotic use post-operation.

## Conclusions

This study shows that there is a gap of good quality data regarding the time to return to work and narcotic use after lumbar spinal fusion operations using MIS or OS techniques. This study also indicates that patients who have lumbar spinal fusion operations with the MIS procedure generally return to work after surgery more quickly and require less post-operation narcotics for pain control compared to patients who had OS. As the societal costs for these patients are likely to be significant, and the availability of studies with high quality is low, it is of great importance for future studies to include data collection regarding time to return to work and narcotic use during the post-operation follow-up period.

## Additional files

Additional file 1: Sample search strategy. The sample search strategy contains all key terms that were searched to identify studies from electronic bibliographic databases. (DOCX 16 kb)

Additional file 2: List of included studies. A list of studies that were finally included after title/abstract and full text evaluations in this SLR. (DOCX 18 kb)

Additional file 3: Quality assessment results using the NICE methodology checklist. This table presents the quality assessment results per included studies using the NICE methodology checklist [65]. (DOCX 85 kb)

Additional file 4: Results of pre-operation vs. post-operation employment rate. This table summarizes the results of pre-operation vs. post-operation employment rate from the eight studies that focused on OS [65]. (DOCX 31 kb)

Additional file 5: Other results of post-operation narcotic usage. This table shows the results of post-operation narcotic uses other than the duration of post-operation narcotic use. (DOCX 59 kb)

## Abbreviations

AAOS: American Academy of Orthopaedic Surgeons
ALIF: Anterior lumbar interbody fusion
AxiaLIF: Axial lumbar interbody fusion
CADTH: Canadian Agency for Drugs and Technologies in Health
CDR: Canadian Agency for Drugs
CRD: Centre for Reviews and Dissemination
DARE: Database of abstracts of reviews of effects
EFFOT: European Federation of National Associations of Orthopaedics and Traumatology
ISASS: International Society for the Advancement of Spine Surgery
ISPOR: International Society for Pharmacoeconomics Outcomes Research
MIS: Minimally invasive surgery
NASS: North American Spine Society
NHS EED: NHS Economic Evaluations Database
NICE: National Institute and Clinical Excellence
NIH: National Institutes of Health
NSAID: Non-Steroidal Anti-Inflammatory Drug
OS: Open surgery
OTA: Orthopaedic Trauma Association
PBAC: Pharmaceutical Benefits Advisory Committee
PLF: Posterolateral fusion
PLIF: Posterior lumbar interbody fusion
RCT: Randomized controlled trial
SMISS: Society for MINIMALLY INVASIVE SPINE SURGERY
TDR: Total disc replacement
TLIF: Transforaminal lumbar interbody fusion
