# Supplementary material for: A systematic literature review of time to return to work and narcotic use after lumbar spinal fusion using minimal invasive and open surgery techniques
Source: BMC Health Serv Res. 2017 Jun 27;17:446. doi: 10.1186/s12913-017-2398-6 (PMC5488344; doi:10.1186/s12913-017-2398-6)
Supplement: Supplementary file 1 — Sample search strategy. The sample search strategy contains all key terms that were searched to identify studies from electronic bibliographic databases. (DOCX 16 kb) [file 12913_2017_2398_MOESM1_ESM.docx]

## Additional file 1: Sample search strategy

| 1 | "Spinal Fusion"[mesh] OR (spinal[text] AND (infusion[text] OR infusions[text])) OR "spinal fusion"[text] OR "spinal fusions"[text] |
| --- | --- |
| 2 | ("lumbar fusion"[text] OR "lumbar fusions"[text]) |
| 3 | ("lumbar interbody fusion"[text] OR "lumbar interbody fusions"[text]) |
| 4 | ("thoracolumbar fusion"[text] OR "thoracolumbar fusions"[text]) |
| 5 | (#1 OR #2 OR #3 OR #4) |
| 6 | "Orthopedic Procedures"[Mesh] |
| 7 | "open surgery"[text] OR "open surgeries" OR "open surgical procedure"[text] or "open surgical procedures"[text] OR "open procedure"[text] OR "open procedures"[text] |
| 8 | "traditional approach"[text] OR "traditional approaches"[text] OR "conventional approach"[text] OR "conventional approaches"[text] |
| 9 | "surgical procedures, minimally invasive"[mesh] |
| 10 | "Surgical Procedures, Minor"[Mesh] |
| 11 | "minimally invasive surgery"[text] OR "minimally invasive surgeries"[text] |
| 12 | "minimally invasive spinal surgery"[text] OR "minimally invasive spinal surgeries"[text] |
| 13 | "minimally invasive"[text] |
| 14 | "minimally invasive approach"[text] OR "minimally invasive approaches"[text] |
| 15 | "percutaneous"[text] |
| 16 | "back pain"[mesh] OR "back pain"[tiab] |
| 17 | "Spinal Diseases"[mesh] |
| 18 | "Intervertebral disc disease" [Supplementary Concept] OR "Intervertebral Disc Degeneration"[text] OR "Intervertebral Disc Displacement"[text] OR "intervertebral disc disease"[text] |
| 19 | "degenerative lumbar disease"[text] |
| 20 | "Isthmic Spondylolisthesis"[text] |
| 21 | "Spondylolisthesis"[text] |
| 22 | ((#6 OR #7 OR #8 OR #9 OR #10 OR #11 OR #12 OR #13 OR #14 OR #15 OR #16 OR #17 OR #18 OR #19 OR #20 OR #21)) |
| 23 | ("Sick Leave"[Mesh] OR ((leave[tiab] or leaves[tiab]) AND Sick[tiab])) |
| 24 | ("medical leave"[text] OR "medical leaves"[text]) |
| 25 | "sickness benefit"[text] OR "sickness benefits"[text] |
| 26 | "Disability Leave"[text] OR "Disability Leaves"[text] OR ( Disability[tiab] AND (leave[tiab] OR leaves[tiab])) |
| 27 | "Sick Days"[text] OR ( sick[tiab] AND (days[tiab] OR day[tiab])) |
| 28 | "Illness Days"[text] OR ( Illness[tiab] AND (days[tiab] OR day[tiab])) |
| 29 | Absenteeism[Mesh] OR Absenteeism[text] OR "absence from work"[text] OR "absent from work"[text] OR ("absence"[text] AND (work[mesh] OR work[text])) |
| 30 | "Return to Work"[Mesh] OR "return to work"[text] OR "return-to-work"[text] |
| 31 | "back to work"[text] OR "back-to-work"[text] |
| 32 | "loss of productivity"[text] |
| 33 | "Work Productivity"[text] OR "WPAI"[text] OR "Work Productivity and Activity Impairment"[text] |
| 34 | ((#23 OR #24 OR #25 OR #26 OR #27 OR #28 OR #29 OR #30 OR #31 OR #32 OR #33)) |
| 35 | "Narcotics"[Mesh] OR "narcotics"[text] |
| 36 | "Analgesics"[Mesh] AND "Analgesics, Non-Narcotic"[Mesh] AND "Analgesics, Short-Acting"[Mesh] AND "Analgesics, Opioid"[Mesh] OR "analgesics"[text] OR "analgesic drugs"[text] OR "analgesic agents"[text] OR "opioids"[text] OR "aspirin"[text] OR "paracetamol"[text] OR "tramadol"[text] OR "antinociceptive"[text] |
| 37 | "Morphinans"[Mesh] OR "morphine"[mesh] OR "morphine"[text] |
| 38 | "Anti-Inflammatory Agents, Non-Steroidal"[mesh] OR "Non-Steroidal Anti-Inflammatory Drug"[text] OR "Non-Steroidal Anti-Inflammatory Drugs"[text] OR "NSAID"[text] OR "NSAIDs"[text] |
| 39 | "Glucocorticoids"[Mesh] OR "corticoids"[text] |
| 40 | "Antidepressive Agents, Tricyclic"[Mesh] OR "antidepressant"[text] OR "antidepressants"[text] |
| 41 | "Anticonvulsants"[Mesh] OR "gabapentin"[text] |
| 42 | ("Muscle Relaxants"[text] OR "Muscle Relaxants, Central"[Mesh] OR "Muscle Relaxant"[text]) |
| 43 | (#35 OR #36 OR #37 OR #38 OR #39 OR #40 OR #41 OR #42) |
| 44 | (#34 OR #43) |
| 45 | #5 AND #22 AND #44 |
| 46 | (animals[mh]) NOT ((animals[ mh]) AND (human[mh])) |
| 47 | english[lang] OR swedish[lang] OR german[lang] |
| 48 | (#45 AND #47) NOT #46 |
| 49 | (#45 AND #47) NOT #46 Filters: Publication date from 2004/01/01 |
