# Supplementary material for: A systematic literature review of time to return to work and narcotic use after lumbar spinal fusion using minimal invasive and open surgery techniques
Source: BMC Health Serv Res. 2017 Jun 27;17:446. doi: 10.1186/s12913-017-2398-6 (PMC5488344; doi:10.1186/s12913-017-2398-6)
Supplement: Supplementary file 2 — List of included studies. A list of studies that were finally included after title/abstract and full text evaluations in this SLR. (DOCX 18 kb) [file 12913_2017_2398_MOESM2_ESM.docx]

## Additional file 2: List of included studies

Adogwa, O., et al. "Comparative effectiveness of minimally invasive versus open transforaminal lumbar interbody fusion: 2-year assessment of narcotic use, return to work, disability, and quality of life." Journal of spinal disorders & techniques 24.8 (2011): 479-84.

Berg, S., et al. "Total disc replacement compared to lumbar fusion: a randomised controlled trial with 2-year follow-up." European spine journal : official publication of the European Spine Society, the European Spinal Deformity Society, and the European Section of the Cervical Spine Research Society 18.10 (2009): 1512-19.

Blumenthal, S., et al. "A prospective, randomized, multicenter Food and Drug Administration investigational device exemptions study of lumbar total disc replacement with the CHARITE artificial disc versus lumbar fusion: part I: evaluation of clinical outcomes." Spine (Phila Pa 1976.) 30.14 (2005): 1565-75.

Bochicchio, M., et al. "Minimally invasive technique with transforaminal interbody fusion (TLIF) and interspinous/interlaminar arthrodesis (IFD). Mid term radiologic and clinical evaluation: Fusion rate? Complications? Real advantages? 26.":907. 2013. Springer Verlag.

Bochicchio, M., et al. "Minimally invasive posterior interspinous and interlaminar fusion device for one level fusion in degenerative lumbar disease: Mid term clinical, and radiological evaluation and complications report 25.":932. 2013. Springer Verlag.

Brotis, A. G., et al. "Instrumented posterior lumbar fusion outcomes for lumbar degenerative disorders in a southern European, semirural population." Journal of spinal disorders & techniques 23.7 (2010): 444-50.

Buttermann, G. R., Thorson, T. M., and Mullin, W. J. "Outcomes of posterior facet versus pedicle screw fixation of circumferential fusion: A cohort study 17.":347-355. 2014. Springer Verlag (Tiergartenstrasse 17, Heidelberg D-69121, Germany).

Cheng, J. S., et al. "Short-term and long-term outcomes of minimally invasive and open transforaminal lumbar interbody fusions: is there a difference?" Neurosurgical focus 35.2 (2013): E6.

Corenman, D. S., et al. "Recombinant human bone morphogenetic protein-2-augmented transforaminal lumbar interbody fusion for the treatment of chronic low back pain secondary to the homogeneous diagnosis of discogenic pain syndrome: two-year outcomes." Spine (Phila Pa 1976.) 38.20 (2013): E1269-E1277.

Eckman, W. W., L. Hester, and M. McMillen. "Same-day discharge after minimally invasive transforaminal lumbar interbody fusion: a series of 808 cases." Clin.Orthop Relat Res. 472.6 (2014): 1806-12.

Eckman, W. W., Hester, L. G., and McMillen, M. "Unilateral Minimally Invasive Transforaminal Lumbar Interbody Fusion (MITLIF): Results of 670 cases discharged the day of surgery 194.":119S. 2012. Elsevier Inc.

Fayssoux, R., et al. "Indirect costs associated with surgery for low back pain-a secondary analysis of clinical trial data." Popul.Health Manag 13.1 (2010): 9-13.

Fritzell, P., et al. "Cost effectiveness of disc prosthesis versus lumbar fusion in patients with chronic low back pain: randomized controlled trial with 2-year follow-up." European spine journal : official publication of the European Spine Society, the European Spinal Deformity Society, and the European Section of the Cervical Spine Research Society 20.7 (2011): 1001-11.

Fritzell, P., et al. "Cost-effectiveness of lumbar fusion and nonsurgical treatment for chronic low back pain in the Swedish Lumbar Spine Study: a multicenter, randomized, controlled trial from the Swedish Lumbar Spine Study Group." Spine (Phila Pa 1976.) 29.4 (2004): 421-34.

Froholdt, A., et al. "No difference in 9-year outcome in CLBP patients randomized to lumbar fusion versus cognitive intervention and exercises." European spine journal : official publication of the European Spine Society, the European Spinal Deformity Society, and the European Section of the Cervical Spine Research Society 21.12 (2012): 2531-38.

Gillard, D. M., D. S. Corenman, and G. J. Dornan. "Failed less invasive lumbar spine surgery as a predictor of subsequent fusion outcomes." Int Orthop 38.4 (2014): 811-15.

Gornet, M. F., et al. "Lumbar disc arthroplasty with Maverick disc versus stand-alone interbody fusion: a prospective, randomized, controlled, multicenter investigational device exemption trial." Spine (Phila Pa 1976.) 36.25 (2011): E1600-E1611.

Guyer, R. D., et al. "Prospective, randomized, multicenter Food and Drug Administration investigational device exemption study of lumbar total disc replacement with the CHARITE artificial disc versus lumbar fusion: five-year follow-up." Spine J 9.5 (2009): 374-86.

Hamid, N., et al. "Percutaneous spinal stabilization-is the future here? 23.":S64. 2013. Springer Verlag.

Isaacs, R. E., et al. "Minimally invasive microendoscopy-assisted transforaminal lumbar interbody fusion with instrumentation." Journal of neurosurgery.Spine 3.2 (2005): 98-105.

Kim, D. Y., et al. "Comparison of multifidus muscle atrophy and trunk extension muscle strength: percutaneous versus open pedicle screw fixation." Spine (Phila Pa 1976.) 30.1 (2005): 123-29.

Kim, J. S., B. Jung, and S. H. Lee. "Instrumented Minimally Invasive spinal-Transforaminal Lumbar Interbody Fusion (MIS-TLIF); Minimum 5-years Follow-up With Clinical and Radiologic Outcomes." Journal of spinal disorders & techniques (2012).

Kim, J. S., et al. "Mini-transforaminal lumbar interbody fusion versus anterior lumbar interbody fusion augmented by percutaneous pedicle screw fixation: a comparison of surgical outcomes in adult low-grade isthmic spondylolisthesis." Journal of spinal disorders & techniques 22.2 (2009): 114-21.

Kim, J.-S., Choi, W. G., and Lee, S.-H. "Minimally invasive anterior lumbar interbody fusion followed by percutaneous pedicle screw fixation for isthmic spondylolisthesis: minimum 5-year follow-up 363.":404-409. 2010. Elsevier Inc. (360 Park Avenue South, New York NY 10010, United States).

Kim, J.-S., et al. "Comparison study of the instrumented circumferential fusion with instrumented anterior lumbar interbody fusion as a surgical procedure for adult low-grade isthmic spondylolisthesis 382.":565-571. 2010. Elsevier Inc. (360 Park Avenue South, New York NY 10010, United States).

Lee, K. H., et al. "Clinical and radiological outcomes of open versus minimally invasive transforminal lumbar interbody fusion 338.":102S-103S. 2011. Elsevier Inc.

Parker, S. L., et al. "Cost-effectiveness of minimally invasive versus open transforaminal lumbar interbody fusion for degenerative spondylolisthesis associated low-back and leg pain over two years." World Neurosurg 78.1-2 (2012): 178-84.

Parker, S. L., et al. "Minimally Invasive versus Open Transforaminal Lumbar Interbody Fusion for Degenerative Spondylolisthesis: Comparative Effectiveness and Cost-Utility Analysis." World Neurosurg (2013).

Potter, B. K., et al. "Transforaminal lumbar interbody fusion: Clinical and radiographic results and complications in 100 consecutive patients 656.":337-346. 2005. Lippincott Williams and Wilkins.

Robertson, P. A. and S. A. Jackson. "Prospective assessment of outcomes improvement following fusion for low back pain." Journal of spinal disorders & techniques 17.3 (2004): 183-88.

Rodriguez-Vela, J., et al. "Perioperative and short-term advantages of mini-open approach for lumbar spinal fusion 472.":1194-1201. 2009. Springer Verlag (Tiergartenstrasse 17, Heidelberg D-69121, Germany).

Rouben, D., M. Casnellie, and M. Ferguson. "Long-term durability of minimal invasive posterior transforaminal lumbar interbody fusion: a clinical and radiographic follow-up." Journal of spinal disorders & techniques 24.5 (2011): 288-96.

Schwender, J. D., et al. "Minimally invasive transforaminal lumbar interbody fusion (TLIF): technical feasibility and initial results 651.":S1-S6. 2005.

Takahashi, T., et al. "Surgical outcome and postoperative work status of lumbar discogenic pain following transforaminal interbody fusion." Neurol.Med Chir (Tokyo) 51.2 (2011): 101-07.

Wenger, M., Sapio, N., and Markwalder, T. M. "Long-term outcome in 132 consecutive patients after posterior internal fixation and fusion for Grade I and II isthmic spondylolisthesis 652.":289-297. 2005.

Zeilstra, D. J., Miller, L. E., and Block, J. E. "Axial lumbar interbody fusion: A 6-year single-center experience 87.":1063-1069. 2013. Dove Medical Press Ltd. (PO Box 300-008, Albany, Auckland, New Zealand) http://www.dovepress.com/getfile.php?fileID=17098.
