# Supplementary material for: A systematic literature review of time to return to work and narcotic use after lumbar spinal fusion using minimal invasive and open surgery techniques
Source: BMC Health Serv Res. 2017 Jun 27;17:446. doi: 10.1186/s12913-017-2398-6 (PMC5488344; doi:10.1186/s12913-017-2398-6)
Supplement: Supplementary file 3 — Quality assessment results using the NICE methodology checklist. This table presents the quality assessment results per included studies using the NICE methodology checklist [65]. (DOCX 85 kb) [file 12913_2017_2398_MOESM3_ESM.docx]

## Additional file 3: Quality assessment results using the NICE methodology checklist

| **Study** | **NICE methodology checklist [**[**20**](#_ENREF_20)**]** | | | | | |
| --- | --- | --- | --- | --- | --- | --- |
|  | **Population** | **Method** | **Outcomes** | **Analyses** | **Summary** | **Overall assessment** |
| Adogwa et al. (2011) [[21](#_ENREF_21)] | + | - | + | + | + | **+** |
| Berg et al. (2009) [[36](#_ENREF_36)] | + | + | + | + | + | **+** |
| Blumenthal et al. (2005) [[38](#_ENREF_38)] | ++ | ++ | + | + | + | **++** |
| Bochicchio et al. (2013) [[41](#_ENREF_41)] | - | - | - | - | - | **-** |
| Bochicchio et al. (2013) [[42](#_ENREF_42)] | - | - | - | - | - | **-** |
| Brotis et al. (2010) [[56](#_ENREF_56)] | - | - | - | - | - | **-** |
| Buttermann et al. (2014) [[44](#_ENREF_44)] | + | - | + | - | + | **+** |
| Cheng et al. (2013) [[50](#_ENREF_50)] | + | + | + | + | + | **+** |
| Corenman et al. (2013) [[37](#_ENREF_37)] | - | - | + | + | - | **-** |
| Eckman et al. (2012) [[32](#_ENREF_32)] | - | - | - | - | - | **-** |
| Eckman et al. (2014) [[31](#_ENREF_31)] | - | - | + | + | - | **-** |
| Fayssoux et al. (2010) [[39](#_ENREF_39)] | - | - | - | - | + | **-** |
| Fritzell et al. ( 2004) [[29](#_ENREF_29)] | ++ | ++ | ++ | ++ | ++ | **++** |
| Fritzell et al. (2011) [[30](#_ENREF_30)] | + | + | + | ++ | + | **+** |
| Froholdt et al. (2012) [[45](#_ENREF_45)] | + | - | + | ++ | - | **+** |
| Gillard et al. (2014) [[57](#_ENREF_57)] | - | - | - | - | - | **-** |
| Gornet et al. (2011) [[28](#_ENREF_28)] | + | + | ++ | ++ | + | **++** |
| Guyer et al. (2009) [[40](#_ENREF_40)] | ++ | ++ | + | ++ | + | **++** |
| Hamid et al. (2013) [[46](#_ENREF_46)] | - | - | - | - | - | **-** |
| Isaacs et al. (2005) [[49](#_ENREF_49)] | - | - | - | - | - | **-** |
| Kim et al. (2005) [[51](#_ENREF_51)] | - | - | + | - | + | **-** |
| Kim et al. (2009) [[24](#_ENREF_24)] | - | - | - | + | - | **-** |
| Kim et al. (2010) [[35](#_ENREF_35)] | - | - | - | - | - | **-** |
| Kim et al. (2010) [[23](#_ENREF_23)] | + | - | + | - | + | **+** |
| Kim et al. (2012) [[34](#_ENREF_34)] | - | - | - | - | + | **-** |
| Lee et al. (2011) [[43](#_ENREF_43)] | - | - | - | - | - | **-** |
| Parker et al. (2012) [[18](#_ENREF_18)] | + | - | + | + | + | **+** |
| Parker et al. (2013) [[22](#_ENREF_22)] | + | - | + | + | + | **+** |
| Potter et al. (2005) [[47](#_ENREF_47)] | - | - | + | + | + | **+** |
| Robertson et al. (2004) [[26](#_ENREF_26)] | - | - | - | - | + | **-** |
| Rodriguez-Vela et al. (2009) [[48](#_ENREF_48)] | - | - | - | - | - | **-** |
| Rouben et al. (2011) [[25](#_ENREF_25)] | + | - | + | + | + | **+** |
| Schwender et al. (2005) [[52](#_ENREF_52)] | - | - | - | - | - | **-** |
| Takahashi et al. (2011) [[27](#_ENREF_27)] | - | - | - | + | + | **-** |
| Wenger et al. (2005) [[65](#_ENREF_65)] | - | - | - | - | - | **-** |
| Zeilstra et al. (2013) [[33](#_ENREF_33)] | + | - | - | - | + | **-** |
