# Supplementary material for: A systematic literature review of time to return to work and narcotic use after lumbar spinal fusion using minimal invasive and open surgery techniques
Source: BMC Health Serv Res. 2017 Jun 27;17:446. doi: 10.1186/s12913-017-2398-6 (PMC5488344; doi:10.1186/s12913-017-2398-6)
Supplement: Supplementary file 4 — Results of pre-operation vs. post-operation employment rate. This table summarizes the results of pre-operation vs. post-operation employment rate from the eight studies that focused on OS [65]. (DOCX 31 kb) [file 12913_2017_2398_MOESM4_ESM.docx]

## Additional file 4: Results of pre-operation vs. post-operation employment rate

| **Study** | **Study Design** | **Study country** | **Follow-up** | **Number of patients** | **Number of fusion levels** | **Age (year)** | **Female (%)** | **Pre-operation employment rate** | **Post-operation employment rate** |
| --- | --- | --- | --- | --- | --- | --- | --- | --- | --- |
| Blumenthal et al. (2005) [[38](#_ENREF_38)] | Prospective randomized multicenter | US | 24 months | OS-ALIF: 99 | 1-level | 39.6 (9.07) [20-60] | 55.6% | Work full- or part-time: 57.6% (n=57). | Working full- or part-time at 12 months: 62.5%; at 24 months: 65%. |
| Brotis et al. (2010) [[56](#_ENREF_56)] | Prospective clinical study | Greece | 3 years | OS: 71 | 1-level: 21 (69%);  2-level: 11 (28%);  Multi-level:7 (18%); | NR | NR | NR | At 3 years: 38.5% (n=15) could work and earn their living, among which 28% (n=11) returned to their earlier jobs. |
| Fayssoux et al. (2010) [[39](#_ENREF_39)] | Cost analysis | US | 2 years | OS-ALIF | 1-level | 39.6 (9.07) | 55.6% | Work full- or part-time: 57%; Patients on payroll: 77%. | Working full- or part-time work at 6 weeks: 24%; at 24 months: ~65%.  Patients on payroll at 6 weeks: 37%. |
| Fritzell et al. (2011) [[30](#_ENREF_30)] | Cost effectiveness study | Sweden | 2 years | OS-PLF: 44; OS-PLIF: 28. | NR | 38.5 (7.8) | 58.0% | Sick leave: 70%; Working full- or part-time: 30%; Blue collar workers; n=37. | At 2 years, 72% working full- or part-time work; among them, 20% had changed to lighter duties. |
| Froholdt et al. (2012) [[45](#_ENREF_45)] | Observational study | Norway | 9 years | OS: 55 | NR | 43 (8.1) | 65.0% | Working: 16% (n=9); Sick leave: 24% (n=13); Rehabilitation: 38% (n=21);  Disability pension: 15% (n=8); Other: 7% (n=4). | At 9 years: 35% (n=19) working full or part-time |
| Gornet et al. (2011) [[28](#_ENREF_28)] | Randomized controlled trial | US | 2 years (91.4% patients) | OS-ALIF: 172 | 1-level | 40.2 [18-65] | 50.0% | Working: 55.8% (n=96); Workers' compensation: 17.4% (n=30). | At 1.5 months: 26% were working; at 3 months: 41.6% were working; at 6 months: 63.4% were working; at 12 months: 66.9% were working; at 24 months: 73.4% were working. |
| Guyer et al. (2009) [[40](#_ENREF_40)] | Prospective randomized multicenter | US | 5 years | OS-ALIF: 43 | 1-level | 38.8 (8.69) [25-55] | 44.0% | Full-time: 49% (n=21); Part-time: 7% (n=3); Short-term disability: 7% (n=7); Long-term disability: 9% (n=4); Not employed: 9% (n=4); Other: 9% (n=8). | At 5 years: working full-time 46.5%; long-term disability: 20.9% |
| Wenger et al. (2005) [[65](#_ENREF_65)] | Retrospective study | Switzerland | 9.9 years (0.5-19.4) | OS-PLF/PLIF: 132 | NR | 40.6 [15.2-69.9] | 50.0% | NR | 93.2% (n=123) described their work status:  - working full-time: n=68; - working part-time: n= 16;  - worker's compensation: n= 3 (1 was unemployed, and 1 was completing a course for job retraining); - on disability compensation or had retired: n=34. |

*ALIF: Anterior Lumbar Interbody Fusion; MIS: Minimal Invasive Surgery; NR: Not Reported; OS: Open Surgery; PLF: PosteroLateral Fusion; PLIF: Posterior Lumbar Interbody Fusion; TLIF: Transforaminal Lumbar Interbody Fusion*
