# Supplementary material for: A systematic literature review of time to return to work and narcotic use after lumbar spinal fusion using minimal invasive and open surgery techniques
Source: BMC Health Serv Res. 2017 Jun 27;17:446. doi: 10.1186/s12913-017-2398-6 (PMC5488344; doi:10.1186/s12913-017-2398-6)
Supplement: Supplementary file 5 — Other results of post-operation narcotic usage. This table shows the results of post-operation narcotic uses other than the duration of post-operation narcotic use. (DOCX 59 kb) [file 12913_2017_2398_MOESM5_ESM.docx]

## Additional file 5: Other results of post-operation narcotic usage

| ***Study*** | ***Study country*** | ***Study Design*** | ***Follow-up*** | ***Number of patients*** | ***Number of fusion levels*** | ***Age (year)*** | ***Female (%)*** | ***Pre- and post-operation narcotic usage*** | | |
| --- | --- | --- | --- | --- | --- | --- | --- | --- | --- | --- |
|  |  |  |  |  |  |  |  | ***MIS*** | ***OPEN*** | ***P-value*** |
| ***MIS vs. OS studies*** | | | | | | | | | | |
| Adogwa et al. (2011) [[21](#_ENREF_21)] | US | Retrospective cohort study | 2 years | 15 (MIS-TLIF) 15 (OS-TLIF) | 1-level | MIS: 50.8 (7.9) OS: 49.7 (11.4) | MIS: 53.3% OS: 66.7% | At 1 month: ~90% discontinued narcotics; At 2 months: ~100% discontinued narcotics. | At 4 month: ~90% discontinued narcotics; At 6 months: ~100% discontinued narcotics. | NR |
| Buttermann et al. (2014) [[44](#_ENREF_44)] | US | Prospective study | 5.6 years | 35 (MIS-posterior instrumentation) 27 (OS-Circumferential spinal fusion) | 1-level | MIS: 38.1 (10.4) OS: 42.4 (12.8) | MIS: 86% OS: 63% | Pre-: 51% (n=18) on narcotics; 49% (n=17) on NSAID. At 7–12 months: 34 % (n=12) on narcotics; 17% (n=6) on NSAID.  At 1–2 years: 34% (n=12) on narcotics; 34 % (n=12) on NSAID. At 2–4 years: 37% (n=13) on narcotics; 40% (n=14) on NSAID. At 4–6 years: 27 %(n=8) on narcotics; 33% (n=10) on NSAID. | Pre-: 59% (n=16) on narcotics; 52% (n=14) on NSAID. At 7–12 months: 63 % (n=17) on narcotics; 22% (n=6) on NSAID.  At 1–2 years: 33% (n=9) on narcotics; 37 % (n=10) on NSAID. At 2–4 years: 41% (n=11) on narcotics; 48% (n=13) on NSAID. At 4–6 years: 28 %(n=7) on narcotics; 48% (n=12) on NSAID. | Narcotics use at 7–12 months: p<0.05. |
| Isaacs et al. (2005) [[49](#_ENREF_49)] | US | Cohort study | NR | 20 (MIS-TLIF) 24 (OS-PLIF) | 1-level | MIS: 47.8 [31-67] OS: 51 | MIS: 65% OS: 33% | Normalized for LOS: 37.5 morphine sulfate equivalents/day. | Normalized for LOS: 49.5 morphine sulfate equivalents/day. | NR |
| Cheng et al. (2013) [[50](#_ENREF_50)] | US | Retrospective chart-review study | 5.05 (1.4) years | 50 (MIS-TLIF) 25 (OS-PLIF) | 1-level | MIS: 53.7 (13.5) OS: 54.3 (11.1) | MIS: 46% OS: 44% | Pre-:  Narcotics around the clock (mg/day): 35.6 (11.2);  narcotics as needed (mg/day): 53.7 (10.6);  28% (n=14) on muscle relaxants. Post-:  76% (n=38) on muscle relaxants; Narcotics around the clock (mg/day): 66.5 (21.9); Narcotics as needed (mg/day): 140.5 (15). | Pre-:  Narcotics around the clock (mg/day): 92.2 (32.2); Narcotics as needed (mg/day): 96.6 (48.3); 16% (n=4) on muscle relaxants. Not significant Post-: 80% (n=20) on muscle relaxants; Narcotics around the clock (mg/day): 201.5 (61.2); Narcotics as needed (mg/day): 153.9 (50.1). | Muscle relaxants: p=0.51; Narcotics around the clock (mg/day): p=0.019; Narcotics as needed (mg/day): p=0.37 |
| Parker et al. (2012) [[18](#_ENREF_18)] | US | Cost-effectiveness Prospective cohort study | 2 years | 15 (MIS-TLIF) 15 (OS-TLIF) | 1-level | MIS: 50.8 (7.9) OS: 49.7 (11.4) | MIS: 53.3% OS: 66.7% | Post-: NSAID: $297 (0–691)  Oral steroids : $35 (7–63)  Narcotics: $97 (62–132) Muscle relaxants: $62 (18–107)  Antidepressants: $189 (0–387)  Injections: $58 (0–138) | Post-: NASID: $791 (270–1311) Oral steroids : $28 (0–60) Narcotics: $245 (153–338) Muscle relaxants: $214 (0–446) Antidepressants : $152 (0–399) Injections: $33 (0–97) | NR |
| Parker et al. (2013) [[22](#_ENREF_22)] | US | Cost-effectiveness and cost utility analysis | 2 years | 50 (MIS-TLIF) 50 (OS-TLIF) | 1-level | MIS: 53.5 (12.5) OS: 52.6 (11.6) | MIS: 68% OS: 64% | At 6 months: ~18% on narcotics; at 24 months: ~15% on narcotics. | At 6 months: ~25% on narcotics; at 24 months: ~20% on narcotics. | NR |
| Hamid et al. (2013)* [[46](#_ENREF_46)] | NR | Prospective study | NR | 46 (MIS-PLIF) 54 (OS-PLIF) | NR | NR | NR | Post-: 45% required morphine | Post-: 85% required morphine | NR |
| Kim et al. (2005) [[51](#_ENREF_51)] | South Korea | Retrospective case selection and prospective observation | MIS: 20.6 months; OS: 21.5 months. | 8 (MIS-ALIF) 5 (OS-PLIF) 6 (OS-ALIF) | NR | MIS: 60.3 [46-76] OS: 52.4 [35-72] | MIS: 62.5% OS: 63.6% | Perioperative analgesic injection (no.)/day: 1.8 (0.9-3.8);  Post-operative oral non-opioid analgesic medication: - unnecessary: n=5 (62.5); - 1-3 months: n=3 (37.5%); | Perioperative analgesic injection (no.)/day: 1.9 (0.3-3.6);  Post-operative oral non-opioid analgesic medication: - unnecessary: n=1 (9.1%); - 1-3 months: n=7 (63.6%); - 3-6 months: n=2 (18.2%); - >12 months: n=1 (9.1%). | Perioperative analgesic injection: not significant |
| Lee et al. (2011)* [[43](#_ENREF_43)] | Singapore | Prospective clinical study | 2 years | 72 (MIS-TLIF) 72 (OS-TLIF) | 1-level | NR | NR | Post-: Morphine: 3.4 mg | Post-: Morphine: 33.5 mg | <0.05 |
| Rodriguez et al. (2009) [[48](#_ENREF_48)] | Spain | Prospective randomized study | 3 months | 15 (MIS-Circumferential (360˚) lumbar or lumbosacral fusion) 16 (OS-Circumferential (360˚) lumbar or lumbosacral fusion) | 1-level | MIS: 34.14 (8.1) OS: 42.06 (0.6) | MIS: 40% OS: 26.7% | At 3 months: 33% (n=5) required morphine; 20% patients required analgesics. Analgesics at 3 months (SF-36 physical scale scores): 0.7 (1.2). | At 3 months: 40% (n=6) required morphine; 53.8% patients required analgesics. Analgesics at 3 months (SF-36 physical scale scores): 1.8 (1.5). | Morphine use: p=0.77; Analgesics at 3 months: p=0.016. |
| ***MIS studies*** | | | | | | | | | | |
| Rouben et al. (2011) [[25](#_ENREF_25)] | US |  | 49 months (36-60) | 169 (MIS-TLIF) | ‘1-level (124) 2-level (45) | 44.5 (10.9) [17–73] | 57% | Pre-:  100% patients used narcotics for pain control; At 6 months: 31% used narcotics for pain control. | - | Preop vs. Postop: p<0.05 |
| Schwender et al. (2005) [[52](#_ENREF_52)] | US |  | 22.6 months (18-28) | MIS-TLIF: 49 | 1-level | [23-80] | 61.20% | Discontinued narcotics between 2 and 4 weeks post-operation. | - | - |
| Zeilstra et al. (2013) [[33](#_ENREF_33)] | US |  | at least 1 year follow-up (mean 21 ± 8 months) | MIS-AxiaLIF: 131 | 1-level | 41 (8) | 67.00% | 60% discontinued analgesics;  18% reported occasional use (1-3/week). | - | - |
| ***OS studies*** | | | | | | | | | | |
| Blumenthal et al. (2005) [[38](#_ENREF_38)] | US | Prospective randomized multicenter | 24 months | OS-ALIF: 99 | 1-level | 39.6 (9.07) [20-60] | 55.60% | - | During follow-up: 85.9% (n=85) used narcotics |  |
| Froholdt et al. (2012) [[45](#_ENREF_45)] | Norway | Observational study | 9 years | OS-PLF: 55 | NR | 43 (8.1) | 65.00% | - | At 9 years: 44% patients took pain medication daily or weekly. |  |
| Gornet et al. (2011) [[28](#_ENREF_28)] | US | Randomized controlled multicenter investigational device exemption trial | 2 years (91.4% patients) | OS: 172 | 1-level | 40.2 (18-65) | 50.00% | - | Pre-:  Non-narcotic: 61% (n=105); Weak narcotic: 52% (n=89); Strong narcotic: 30.8% (n=53); Muscle relaxant 42.4% (n=73). |  |
| Potter et al. (2005) [[47](#_ENREF_47)] | US | Retrospective review | 38 months (24-61) | OS-TLIF: 100 | 1-level (64) 2-level (33) 3-level (2) 4-level (1) | 38 (18-72) | 31.00% | - | Pre-: 63% (n=52) reported narcotic use for pain control. Post-:  29% (n=24) reported narcotic use for pain control; 71% (n= 58) reported narcotic-free at follow-up; | Preop vs. Postop: p<0.0001. |
| Robertson et al. (2004) [[26](#_ENREF_26)] | New Zealand | Prospective observational study | 31 months (14-63) | OS-PLF / PLIF: 35 | ‘1-level (12) 2-level (16) | 43 (25-58) | 68.60% | - | Pre-operative pain medication usage:  - NSAID: n=19  - Opiates: n=8  - Tricyclic antidepressant): n=8  - Bbenzodiazepine): n=6  - Simple analgesics: n=17  - Nondepolarizing: n=4  - Muscle relaxants Nil: n=2 ; Post-operative pain medication usage:  - NSAID: n= 5 - Opiates: n= 3 - Tricyclic antidepressant: n= 1 - Benzodiazepines: n= 1 - Simple analgesics: n= 15 - Nondepolarizing: n= 1 - Muscle relaxants Nil: n= 9 |  |

**Published as an abstract.*

*"360 procedure": Posterolateral fusion plus internal fixation with the variable screw placement device plus interbody fusion; ALIF: Anterior Lumbar Interbody Fusion; AxiaLIF: Axial Lumbar Interbody Fusion; MIS: Minimal Invasive Surgery; NR: Not Reported; NSAID: Non-Steroidal Anti-Inflammatory Drug; OS: Open Surgery; PLF: PosteroLateral Fusion; PLIF: Posterior Lumbar Interbody Fusion; PSG: Prior Surgery Group; TLIF: Transforaminal Lumbar Interbody Fusion; XLIF: Extreme Lateral Interbody Fusion*
